# *Mycobacterium genavense* infections in immunocompromised patients with HIV: A clinical case report

**DOI:** 10.1002/ccr3.8993

**Published:** 2024-06-02

**Authors:** Seyed Ali Dehghan Manshadi, Mitra Rezaei, Maryam Moradi, Marjan Hemmatian

**Affiliations:** ^1^ Department of Infectious Disease and Tropical Medicine/Iranian Research Center HIV/AIDS (IRCHA) Tehran University of Medical Science Tehran Iran; ^2^ Genomic Research Center Shahid Beheshti University of Medical Science Tehran Iran; ^3^ Eye Research Center, The Five Senses Health Institute, Rassoul Akram Hospital Iran University of Medical Sciences Tehran Iran

**Keywords:** HIV, immunocompromised patients, *Mycobacterium genavense*, nontuberculous mycobacterium

## Abstract

**Key Clinical Message:**

It is essential to consider non‐tuberculosis mycobacterium in HIV‐positive patients with fever, abdominal pain, weight loss, and splenomegaly.

**Abstract:**

*Mycobacterium genavense* is an opportunistic slow‐growing nontuberculous mycobacterium in patients with immunocompromised backgrounds, especially HIV‐positive patients. In this study, we present two cases of *Mycobacterium genovese* infection in HIV‐positive patients with a good clinical response to accurate treatment.

## 1 INTRODUCTION

*Mycobacterium genavense* is an opportunistic slow‐growing mycobacterium in patients with immunocompromised backgrounds, especially HIV‐positive patients. It was first discovered in an HIV‐positive patient in Geneva, Switzerland.^1^, ^2^

It can manifest as fever, night sweats, and weight loss along with other symptoms such as abdominal pain, nausea, vomiting, and diarrhea when the gastrointestinal tract is involved. The symptoms are similar to those with Mycobacterium avium complex (MAC) disseminated infections.^3^, ^4^ Smear examination from sterile sites such as blood, bone marrow, lymph nodes, and spleen and also blood culture are necessary for the diagnosis.^5^, ^6^ Early diagnosis and proper treatment with rifampin plus another antituberculosis drug such as clarithromycin or doxycycline is essential.^7^ Despite combinational therapy, the overall mortality is high, especially in HIV‐positive patients however, patients who receive macrolide‐containing regimens are believed to have a better prognosis.^8^

Due to the rare presentation of *M. genovese*, the diagnostic and therapeutic challenges we present two cases of HIV‐positive patients with *M. genovese* infection and their clinical process. This study is the first report of HIV‐positive *M. genovese* infection in Iran.

## 2 CASE 1

### 2.1 Case history/examination

A 26‐year‐old male without any history of previous medical problems was admitted to the Imam Khomeini Medical Complex, Tehran, Iran with fever, abdominal pain, nausea, vomiting, and non‐bloody diarrhea for a month after several outpatient therapies. He has had a severe weight loss of about 30 kg in the last 5 years without any dietary problems. His abdominal pain was periumbilical concomitant with nausea, vomiting, and anorexia. The abdominal pain radiated to the back and escalated with food consumption.

In his social history, the patient reported one time of unprotected sexual intercourse about 7 years ago and an opioid addiction for 10 years.

On physical examination, he was severely pale, had a temperature of 38°C, and diffuse oral candidiasis. Epigastric and periumbilical tenderness along with mild splenomegaly was detected during abdominal examination.

### 2.2 Methods

Primary laboratory test results are in favor of positive HIV along with pancytopenia (Tables 1 and 2). Positive HIV was confirmed with enzyme‐linked immunosorbent assay (ELISA).

**TABLE 1:** Laboratory test results.

WBC	**1500** (66% of PMN and 24% of lymphocyte) (4500 to 11,000 WBCs per microliter)^a^	Na	138 (135–145 mEq/L)	ALT	29 (29 to 33 IU/L)
HB	**10.3** (14 to 18 g/dL)	K	4.5 (3.6–5.2 mmol/L)	AST	25 (8 to 33 U/L)
PLT	**80,000** (150,000 to 450,000 platelets per microliter of blood)	Mg	2.1 (1.7 to 2.2 mg/dL)	ALP	**888** (44 to 147 IU/L)
BUN	**25** (5 to 20 mg/dL)	P	4 (2.8 to 4.5 mg/dL)	LDH	**414** (140 to 280 U/L)
Cr	0.9 (0.7 to 1.3 mg/dL)	Ca	8.6 (8.6 to 10.3 mg/dL)	Direct Bilirubin	**0.8** (<0.3 mg/dL)
FBS	85 (70–100 mg/dL)	Albumin	3.4 (3.4 to 5.4 g/dL)	Total Bilirubin	**1.8** (0.1 to 1.2 mg/dL)
PT	12.3 (10 to 13 s)	Uric acid	4.9 (3.5 and 7.2 mg/dL)	GGT	**90** (Normal value of <49)
PTT	31 (25 to 35 s)	ESR	**120** (<15 mm/h)	Amylase and Lipase	Normal
INR	1.12 (2.0 to 3.0)	CRP	**95** (0.3 to 1.0 mg/dL)	IGRA	Negative

*Note:* The bold and underlined values presented in Tables are those with abnormal values.

Abbreviations: ALP, alkaline phosphatase; ALT, alanine aminotransferase; AST, aspartate aminotransferase; BUN, blood urea nitrogen; Ca, calcium; Cr, creatinine; CRP, C‐reactive protein; ESR, erythrocyte sedimentation rate; FBS, fasting blood sugar; GGT, gamma‐glutamyl transpeptidase; HB, hemoglobin; IGRA, interferon gamma release assay; INR, international normalized ratio; K, potassium; LDH, lactate dehydrogenase; Mg, magnesium; Na, sodium; P, phosphorus; PLT, platelet count; PT, prothrombin time; PTT, partial thromboplastin time; WBC, white blood cell.

^a^ Normal ranges are provided within the parenthesis.

**TABLE 2:** Laboratory test results.

HIV Ag and Ab	Positive	B/C	Negative
HBS Ag and Ab	Negative	U/C	Negative
HBC Ag and Ab	Negative	Wright, 2ME, Coombs Wright	Negative
HIV viral load	2038 copy/mL	Toxoplasma IgG	Negative
CD4	34	CMV IgG	Negative

*Note:* Bold values represent HIV viral Load: Undetectable Viral Load is less than 200 copies/mL; CD4 normal range is from 500 to 1400 cells/mm^3^ blood.

Abbreviations: 2ME, 2‐mercaptoethanol Brucella agglutination test; Ab, antibody; Ag, antigen; B/C, blood culture; CD4, clusters of differentiation 4; CMV, cytomegalovirus; HBC, hepatitis B core; HBS, hepatitis B surface; HIV, human immunodeficiency virus; U/C, urine culture.

**FIGURE 1:**
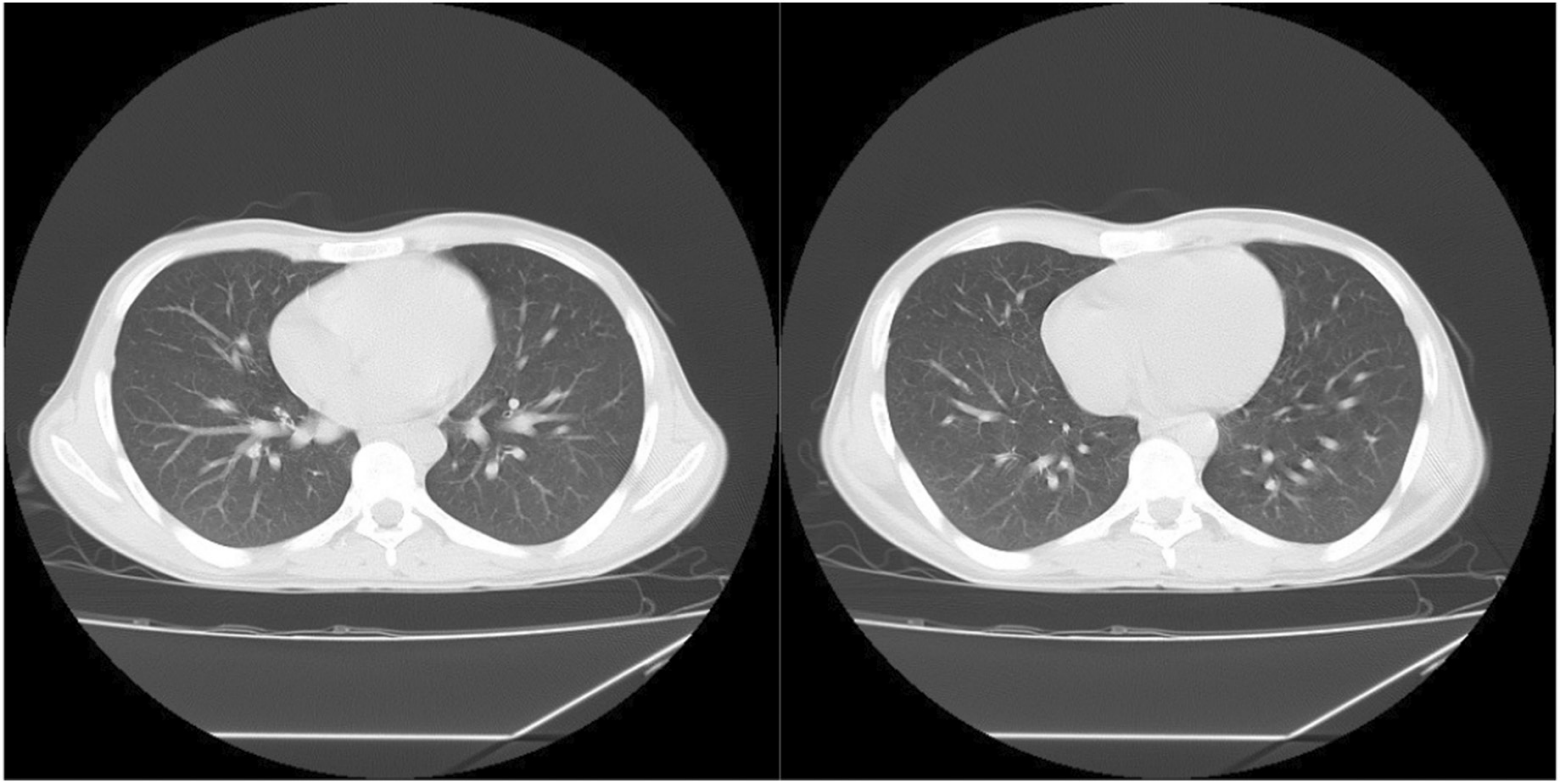
Chest CT scan. An 8‐mm subpleural nodule was observed in the lateral segment of the lower lobe of the right lung (RLL).

**FIGURE 2:**
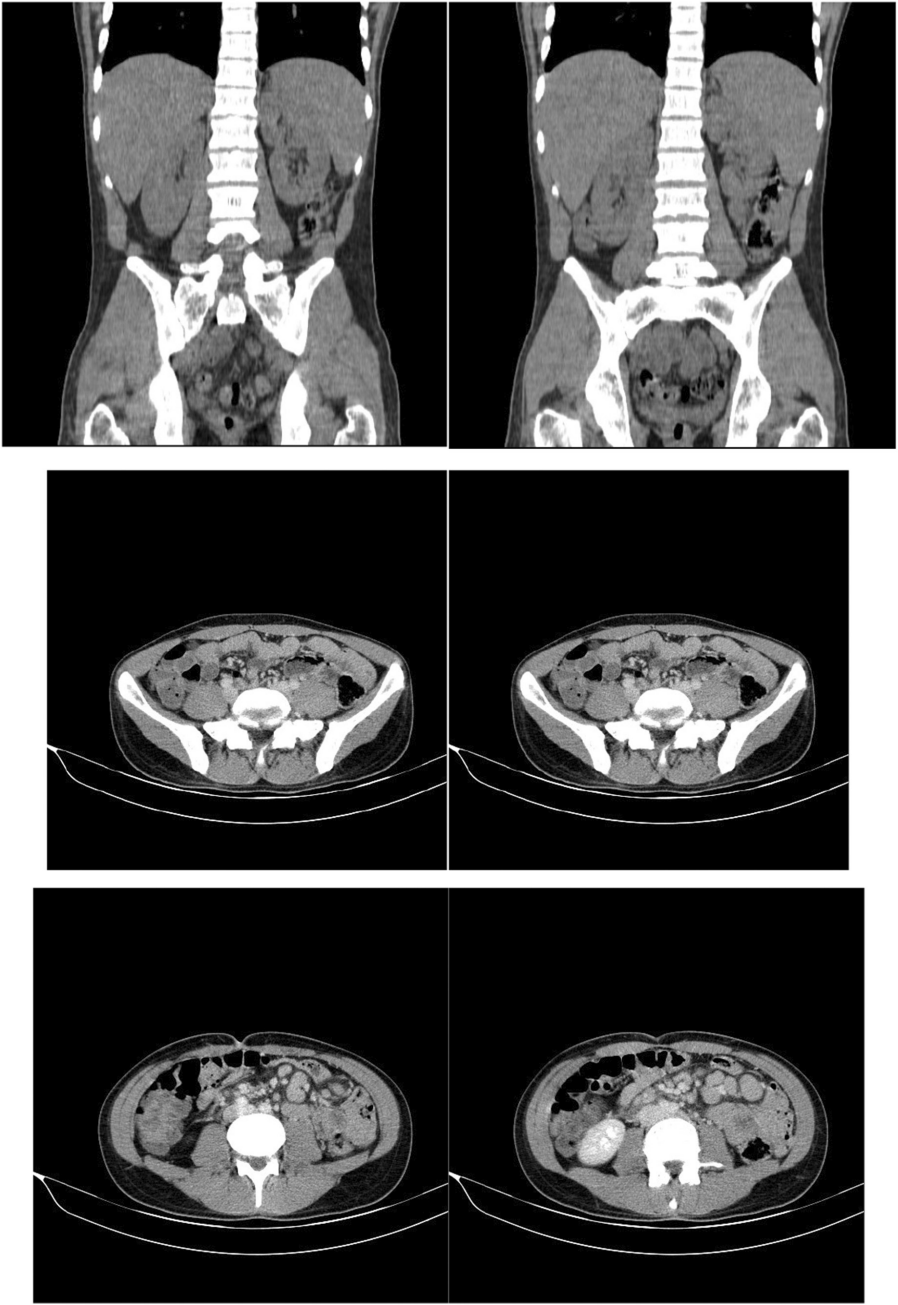
Abdomio‐pelvic CT scan. Mild splenomegaly (150 mm) and multiple mesenteric lymphadenopathies, with a conglomeration pattern and maximum size of 17 mm were detected.

Sputum smear, culture, and polymerase chain reaction (PCR) were negative for Mycobacterium tuberculosis. According to severe abdominal pain, surgical consultation was done. After acute surgical and medical abdomen were ruled out, abdominopelvic sonography and CT scan were performed (Figures 1 and 2). Cryptosporidium was positive in stool examination and esophageal candidiasis along with erythematous mucosal tissue were detected during endoscopic and colonoscopic examinations.

According to the positive HIV tests, non‐bloody diarrhea, splenomegaly, pancytopenia, mesenteric lymphadenopathies, Mycobacterium tuberculosis, atypical mycobacterial infections, and lymphoma were suspected. Bone marrow aspiration and biopsy showed hypocellularity which could be explained according to the positive HIV condition. The Mycobacterium and non‐mycobacterial tuberculosis bone marrow PCRs were all negative.

### 2.3 Conclusion and results

Considering the negative results of bone marrow culture and PCR, a CT scan‐guided biopsy of mesenteric lymph nodes was obtained. According to the positive PCR for *M. genovese*, rifabutin 300 mg/daily, ethambutol 800 mg/daily, azithromycin 500 mg/daily, levofloxacin 500 mg/daily, and amikacin 500 mg/q12h were prescribed. Additional treatments include fluconazole 400 mg /daily for oral candidiasis, TRUVADA (combination of emtricitabine and tenofovir disoproxil fumarate) once daily, and dolutegravir once daily as antiviral agents, and cotrimoxazole as a prophylactic agent were started (these treatments cover both positive HIV and cryptosporidium).

About 10 days after starting the effective treatment, the fever and abdominal pain disappeared, and the patient was discharged with azithromycin 500 mg, ethambutol 800 mg, levofloxacin 750 mg, and rifabutin 300 mg once a day, and the continuation of the antiviral drugs, as well as cotrimoxazole for about 1 year.

## 3 CASE 2

### 3.1 Case history/examination

A 35‐year‐old intravenous drug user male without any history of previous medical problems was admitted to the Imam Khomeini Medical Complex, Tehran, Iran with fever, weight loss, weakness, dysphasia, and odynophagia for 2 months. He has had periodic exertional dyspnea, night fever, and epigastric non‐positional abdominal pain without any radiation. In his social history, he reported methadone and methamphetamine addiction.

On physical examination, he was severely pale, cachexic, had a temperature of 39°C, and diffuse oral and pharyngeal candidiasis. Mild hepatosplenomegaly was detected during abdominal examination which was confirmed by abdominal sonography.

### 3.2 Methods

**FIGURE 3:**
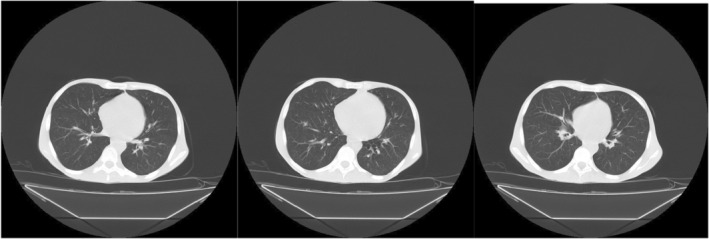
Chest CT scan. There was no abnormal finding.

**FIGURE 4:**
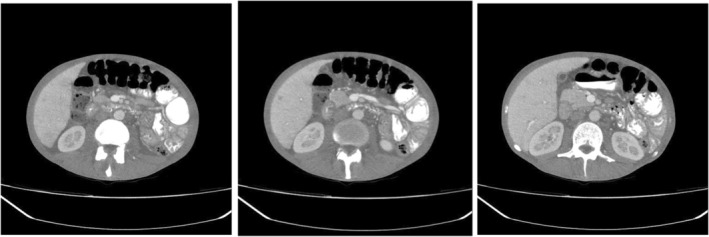
Abdominal CT scan. Hepatomegaly (hepatic span of 170 mm in midclavicular line) with heterogeneous enhancement and periportal edema were seen. An 8‐mm hypodense lesion with peripheral enhancement was observed in the anterior part of the sixth segment of the liver. Mild splenomegaly was seen (135 mm). Multiple mesenteric lymphadenopathies were also detected.

Considering the patient's high‐risk behaviors and oral candidiasis, a rapid HIV test was done. The positive rapid HIV test was confirmed using ELISA. The patient's primary laboratory test results are summarized in Tables 3 and 4. Intravenous fluconazole was started and the candidiasis, dysphagia, and odynophagia were dissolved after 72 h. Endoscopic and colonoscopic examinations were in favor of candidiasis esophagitis. Further evaluations with an abdominopelvic CT scan were done (Figures 3 and 4).

**TABLE 3:** Laboratory test results.

WBC	**3800** (41% of PMN and 52% of lymphocyte) (4500 to 11,000 WBCs per microliter)^a^	Na	**129** (135–145 mEq/L)	ALT	**40** (29 to 33 IU/L)
HB	**6.1** (14 to 18 g/dL)	K	3.8 (3.6–5.2 mmol/L)	AST	**62** (8 to 33 U/L)
PLT	**151,000** (150,000 to 450,000 platelets per microliter of blood)	Mg	2.2 (1.7 to 2.2 mg/dL)	Alk. P	**1102** (44 to 147 IU/L)
BUN	35 (5 to 20 mg/dL)	P	3 (2.8 to 4.5 mg/dL)	LDH	177 (140 to 280 U/L)
Cr	0.7 (0.7 to 1.3 mg/dL)	Ca	8.6 (8.6 to 10.3 mg/dL)	Direct bilirubin	**0.4** (<0.3 mg/dL)
FBS	**120** (70–100 mg/dL)	Albumin	**2.1** (3.4 to 5.4 g/dL)	Total bilirubin	1.1 (0.1 to 1.2 mg/dL)
PT	**16.8** (10 to 13 s)	Uric acid	3.6 (3.5 and 7.2 mg/dL)	GGT	**150** (Normal value of <49)
PTT	30 (25 to 35 s)	ESR	**36** (<15 mm/h)	Amylase and lipase	Normal
INR	1.5 (2.0 to 3.0)	CRP	**9** (0.3 to 1.0 mg/dL)	IGRA	Negative

*Note:* The bold and underlined values presented in Tables are those with abnormal values.

Abbreviations: ALP, alkaline phosphatase; ALT, alanine aminotransferase; AST, aspartate aminotransferase; BUN, blood urea nitrogen; Ca, calcium; Cr, creatinine; CRP, C‐reactive protein; ESR, erythrocyte sedimentation rate; FBS, fasting blood sugar; GGT, gamma‐glutamyl transpeptidase; HB, hemoglobin; IGRA, interferon gamma release assay; INR, international normalized ratio; K, potassium; LDH, lactate dehydrogenase; Mg, magnesium; Na, sodium; P, phosphorus; PLT, platelet count; PT, prothrombin time; PTT, partial thromboplastin time; WBC, white blood cell.

^a^ Normal ranges are provided within the parenthesis.

**TABLE 4:** Laboratory test results.

HIV Ag and Ab	Positive	B/C	Negative
HBS Ag and Ab	Negative	U/C	Negative
HBC Ag and Ab	Negative	Wright, 2ME, Coombs Wright	Negative
HIV viral load	464,357 COPY/ML	Toxoplasma IgG	Positive
CD4	2	CMV IgG	Positive

*Note:* Bold values represent HIV viral Load: Undetectable Viral Load is less than 200 copies/mL; CD4 normal range is from 500 to 1400 cells/mm^3^ blood.

Abbreviations: 2ME, 2‐mercaptoethanol Brucella agglutination test; Ab, antibody; Ag, antigen; B/C, blood culture; CD4, clusters of differentiation 4; CMV, cytomegalovirus; HBC, hepatitis B core; HBS, hepatitis B surface; HIV, human immunodeficiency virus; U/C, urine culture.

According to fever, bicytopenia, hepatosplenomegaly, and positive HIV results, Mycobacterium, non‐mycobacterium tuberculosis, and also lymphoma were suspected. Along with liver and bone marrow biopsy, the 4‐drug “HREZ” fixed‐dose combination with isoniazid (H)–rifampicin (R)–ethambutol (E)–pyrazinamide (Z) plus levofloxacin, TRUVADA and dolutegravir as antiviral agents were started. Based on liver biopsy *M. genavense* type 1 was detected. Despite the expectation, bone marrow PCR was negative for *M. genavense*.

### 3.3 Conclusion and results

Based on the liver biopsy results, anti‐TB drugs were discontinued and the patient was given azithromycin 500 mg, levofloxacin 750 mg, rifabutin 300 mg, and ethambutol 800 mg once a day. After 2 weeks of treatment, fever and abdominal pain were dissolved and the patient was discharged with azithromycin, rifabutin, lofloxacin, ethambutol, and the continuation of the antiviral drugs. Anti‐TB treatment was started in this patient due to prolonged fever, and severe abdominal pain and due to the long process of preparing the biopsy and PCR results, and the treatments were changed when the results were prepared and the diagnosis was determined.

### 3.4 Discussion

*Mycobacterium genavense* is an opportunistic slow‐growing non‐tuberculous mycobacterium affecting patients with underlying immunosuppressive disorders, especially HIV‐positive patients with CD4 counts <100. Its pathogenicity is similar to MAC.^9^, ^10^ Other immunocompromised patients such as solid organ and hematopoietic stem cell recipients, patients with sarcoidosis, and patients with chronic steroid or other immunomodulator drug use are in danger of disseminated *M. genavense* infection.^11^, ^12^ In this case report, we present two male HIV‐positive patients who were diagnosed with *M. genavense* infection.

Based on the meta‐analysis by Wetzstein et al. 76.7% of the patients with *M. genavense* infection were HIV‐positive, and 79.8% were male.^8^ Men are believed to have a higher probability of *M. genavense* infection due to a higher proportion of males among individuals with HIV infection.^10^

The combination therapy with azithromycin, levofloxacin, amikacin, rifabutin, and ethambutol is essential to overcome *M. genavense* infection in patients with or without HIV. Good clinical and radiological treatment responses were seen after the proper treatment. In our patients, after 2 weeks of therapy, the patients were discharged with improved symptoms and acceptable clinical conditions.^13^

In recent studies in our country, HIV‐positive patients with fever, abdominal pain, splenomegaly, and weight loss were considered to have TB infection. Due to the similarity of the treatment choices and the *M. genavense* relative response to TB therapy, the diagnosis was not distinguished. However; it is important to consider non‐tuberculosis mycobacterium such as *M. genavense* in HIV‐positive patients with mentioned symptoms and positive Ziehl–Neelsen stain and start specific treatments.^14^ Based on our knowledge this study was the first case report on *M. genavense* in HIV‐positive patients in Iran.

In conclusion, our study was the first report demonstrating *M. genavense* infection among HIV‐positive patients in Iran. It is essential to consider non‐tuberculosis mycobacterium in HIV‐positive patients with fever, abdominal pain, weight loss, and splenomegaly. Timely diagnosis and proper treatment will reduce mortality and morbidity rates.

## AUTHOR CONTRIBUTIONS

**Seyed Ali Dehghan Manshadi:** Supervision; validation. **Mitra Rezaei:** Validation. **Maryam Moradi:** Writing – original draft. **Marjan Hemmatian:** Resources; supervision; validation.

## FUNDING INFORMATION

None.

## CONFLICT OF INTEREST STATEMENT

The authors have no conflict of interest to declare.

## CONSENT

Written informed consent was obtained from the patient to publish this report in accordance with the journal's patient consent policy.

## Data Availability

The authors confirm that the data supporting the findings of this study are available within the article.
